# International Medical Congress

**Published:** 1876-10

**Authors:** 


### INTERNATIONAL MEDICAL CONGRESS.

### SECTION OF OBSTETRICS.

*Monday, September 4th*, 1876.—Dr. Robert Barnes, of London, chairman, was introduced by Prof. William Goodell. He paid a graceful tribute to the American medical profession, and to American gynæcologists, who had done so much towards making obstetrics an exact science.

### NON-PUERPERAL HEMORRHAGES.

A paper on "The Causes and Treatment of Non-puerperal Hemorrhages of the Womb" was read by Prof. William H. Byford, of Chicago, reporter, ordered to be published, and a vote of thanks tendered the author.

He described the uterus as naturally a hemorrhagic organ; the menstrual changes depending on the ovarian reflex nervous influence exerted unremittingly through the genito-spinal centre. These changes consist of hypertrophy at the commencement of the menstrual flow, denudation of the mucous membrane, and the involutions of that period. He referred at length to the various constitutional and local causes producing disturbances of harmony and profuse flow as a result, and afterwards described the treatment, which is curative, removing the pathological conditions; palliative, such as will stay the bleeding until radical treatment can have its effect.

Prof. Goodell called attention to the inertness, in these cases, of pure astringents, with one exception, when administered by the mouth. He thought the chief good from the use of acetate of lead was through the opium that generally accompanied it. The exception is gallic acid, which he had found very effective (grs. xx.–xxx. every hour). He had given as much as half an ounce through the day (in treacle or jelly.) In a few cases ergot increased the bleeding in some unaccountable way, but in most instances it proved valuable, especially hypodermically. Free dilatation of the cervix is often sufficient to check bleeding; after removing the sponge-tent he applies chemically pure nitric acid, by a swab, to the whole interior surface of the womb, with excellent results. He indorsed Prof. Byford's recommendation of bichloride of mercury in the general treatment, and thought the result was not wholly due to its alterative effect, but largely to its power, in small doses, of increasing the number of red corpuscles, thus correcting the anemia generally present. During hemorrhage he applies heat to the dorso-lumbar region, and gives internally cinnamon tea, or cinnamon boiled in milk, on purely empirical grounds.

Dr. J. W. Rosebrugh, of Hamilton, Canada, thought Prof. Goodell had failed with the acetate because his doses were too small. Dr. Workman, of Toronto, gives it in post-partum hemorrhage in doses of a half to two drachms, and claims that he can relieve any such case by two or three doses. It is a valuable remedy in an emergency.

Dr. Edward M. Hodder, of Toronto, thought the discussion should be restricted to non-puerperal hemorrhages. He uses internally tr. cinnamon, acid sulphuric, and tr. cimicifuga, with local applications of dilute Monsel's solution by means of a glass tube directly to the uterine interior.

Prof. Alex. R. Simpson, of Edinburgh, considered the uterus as "uncertain, coy, and hard *to treat;*" no treatment would suit all cases. Oxide of zinc he had found to materially reduce the menorrhagia (gr. i., thrice daily, or gr. ss. oftener). The later may return from force of habit after the uterus is restored to a healthy condition, especially in hyperæmic cases. He has no great faith in the vaginal tampon as a direct preventive of uterine hemorrhage, but it is sometimes beneficial by the impression and confidence it gives the patient. The sponge-tent is preferred generally, as it opens the way for subsequent exploration and treatment. Nitric acid may be applied in the intervals, but in actual hemorrhage he prefers the perchloride or persulphate of iron. In obscure cases and where the mucous membrane is granular, Prof. James Y. Simpson's plan of scraping off the mucous membrane with a curette proves effective ; he had seen cases cured by it without astringents.

Dr. Thos. W. Gordon, of Ohio, recommended building up the system and full doses of quinia.

Dr. H. F. Campbell, of Georgia, had noticed a periodical character in the bleeding in malarial regions, and uses full doses of quinia. The uterus being engorged from malposition, he insists upon its nightly self-replacement by the patient as indispensable. He uses large cold-water injections between the menstrual periods, with good results.

Dr. Thos. F. Rochester, of Buffalo, thought quinia had an ectrotic influence apart from its anti-periodic power, being valuable in regions non-malarial.

Dr. B. F. Sherman, of New York, recommended Sims's styptic cotton for a tampon, and in granulations a saturated solution of permanganate of potassa. In obscure cases he gives internally oxide of zinc with opium (gr. i. to gr. ¼).

Dr. Chas. Shepard, of Michigan, had checked hemorrhage by injecting ice-water through a female catheter into an india-rubber bag or condom, which, having been previously carried up through the cervix, applied itself closely to the uterine interior.

Dr. Rosebrugh thought gallic acid the only reliable astringent internally. Locally, Monsel's solution is applied *guttatim*, through a glass tube converted into a syringe, by placing a rubber ball at its end.

Dr. Robt. Burns, of Philadelphia, gives tonics, and applies locally a solution of a drachm each of tannic acid and sulphate of zinc in an ounce of glycerine, with excellent results.

Prof. Theophilus Parvin referred with commendation to Trousseau's plan of injecting quantities of hot water, and applying heat to the spine. Hemorrhage, generally accompanying endometritis, can be relieved by scraping or pinching off portions of mucous membrane with forceps, and applying Churchill's tr. of iodine.

The Chairman referred to the mutual advantages of meetings of this kind, where men met to express and compare their views of disease and methods of treatment, and profit by each other's experience. To review remedies and expedients recommended, he would say, first, that quinine was very valuable in the treatment of metorrhagia, especially with sub-involution. He had noticed the law of habit mentioned by Prof. Simpson, in hemorrhages where the uterus was healthy, occurring independently of ovulation and often post-climacteric. They were to be attributed largely to regional attraction of blood to the uterus. Attention must be paid to the constitutional state, especially where no local lesion can be detected. Topically, the most efficacious plan is to dilate the cervix and inject astringents. Gallic acid had succeeded in his hands given by the mouth ; but remedies not strictly astringents are useful, such as digitalis, reducing the heart's action and sometimes checking the bleeding. He had also good results from witch-hazel. The curette treatment should not be adopted in malignant disease, as it may be followed by furious bleeding. It should be handled with great care.

Prof. Byford said that in the vast majority of cases, hemorrhage from a non-pregnant uterus is due to one of two conditions: 1. Hyperemia. The treatment should aim at condensation of uterine structures, and here quinia is second only to ergot. Sponge-tent also induces condensation by pressure. 2. Vitiated state of mucous membrane from inflamation or growths, this structure giving way more readily under the menstrual molimen and bleeding more freely; topical applications are indispensable. He had also noticed a periodic character in metorrhagia in malarial districts. Where bleeding occurs after menopause from determination of blood to the womb, better results had been obtained by him from large doses of quinia and iron than from ergot. The tampon when used should not simply be placed against the os, but packed in front and behind to compress the cervix; he had never witnessed any bad results from its use.

### LABOR IN NARROW PELVES.

*Tuesday, September 5th.*—Dr. Barnes in the chair. Prof. Wm. Goodell, reporter, read his paper on "The Mechanism of Natural *v.* Artificial Labor in Narrow Pelves."

After defining a narrow pelvis, and describing the more common kinds of pelvic deformity, the following topics, regarding alone the mechanism of labor, were introduced for discussion by Dr. Goodell: The mode in which the head enters and passes the brim in the flat pelvis; and also in the generally contracted pelvis, the commonly accepted doctrine of the initial flexion of the head being contested; the manner in which the after-coming head behaves in the flat pelvis; and also in the generally contracted pelvis; the effect instrumental interference has on the mechanism of labor in such pelvis; the inquiry whether turning has any mechanical advantages over the use of the forceps; with the following general conclusions deduced from a consideration of these questions, the scope of the paper being strictly limited to the mechanism of labor in narrow pelves:

1st. The unaided first-coming head and aided after-coming head observe in a flat pelvis precisely the same general laws of engagement and of descent. Hence, version here means art *plus* nature.

2d. The forceps, however applied in a flat pelvis, antagonizes more or less with the natural mechanism of labor. Hence, the forceps here means art *versus* nature.

3d. The aided and unaided first-coming head observe in a uniformly narrowed pelvis precisely the same laws of engagement and of descent. Version violates these laws. Hence, the forceps here means art *plus* nature; version, art *versus* nature.

4th. At or above the brim of a flat pelvis, fronto-mastoid or even fronto-occipital application of forceps interferes less with the moulding of the head, and violates natural mechanism of labor less than biparietal application.

5th. In flat pelves, the vectis aids natural mechanism of labor, and therefore meets applications better than the forceps.

The Chairman pronounced this to be the most able summary of the matter yet heard by him. The passage of the fœtus through the pelvis, and the obstructions to its progress from deformities, were not only to be studied from experience, but, as the conditions are mechanical, by *a priori* reasoning, thorough experience must be the test.

Prof. Byford had heretofore recommended the use of the vectis in the fore or after-coming head when above the brim, and applying the forceps in the canal; but, after hearing this able paper, will be inclined to turn, in kidney-shaped pelves, instead of delivering directly.

Prof. Wm. T. Lusk, of New York, does not believe, as stated in the paper, that the forceps tend to produce extension of the head, but that they will bring it down just as they find it at the pelvic brim. He applies the forceps, pulls the head beyond the constriction, then takes them off, allows the patient to come out of the anæsthesia, and reapplies them in the canal. The danger of applying them above the brim is exaggerated, but it would be wrong to pull the head directly down without reapplying them.

Prof. Goodell, in reply to a question from Dr. Squire, of Elmira, whether his remarks applied also to the forceps of

Chassaignac, said he had no experience in their use. It was significant that the French are tending in this direction of mechanical aids to delivery by bands, straps, and pulleys, borrowing expedients from the veterinary surgeon. In France, turning is so rarely performed as to be almost unknown. Drs. Blot and Pajot, of Paris, who practice turning in emergencies, are the only ones, as far as he knows, who oppose the use of these agents in their practice.

Prof. Simpson said that the flat pelvis presents peculiar advantages for version being performed here with the best hope of success, and theoretically the best result would be anticipated from bringing the base of the skull to the pelvic brim. He thought the amount of force applied to deliver the head after version might endanger the neck of the fœtus, and cited Boudin's experiments on the comparative force required in using forceps and by dragging after turning, the forceps being deemed better in uniformly contracted pelves from their compression of the frontal bones. He had himself found it impossible to apply forceps to the sides of the head when above the brim. They can be applied to the occiput and sinciput, and when the head comes into the canal, removed and applied to the sides.

The Chairman had practiced version instead of the forceps where contraction was at all marked. In minor degrees of contraction, forceps aid in elongating the head and assist delivery. The frequency of occipito-posterior positions in contracted pelves enforces Dr. Goodell's arguments. He cited a case of difficulty at the brim, attended by him in five successive labors, the position in all being occipito-posterior. Another case had some contraction ; the first child was delivered by forceps dead ; in the succeeding two labors, version being resorted to, the children were born alive.

The vectis might be used in minor degrees of contraction where forceps would do as well or nearly as well, but between the forceps and version the vectis does not come in. In version the great danger to the child is in the amount of traction employed. This risk may be reduced by pulling in the line of axis of the superior strait, so that the head may rotate around the projecting promontory as a centre, and by having an assistant push the head backwards with his hand in the hypogastric region. He has seen a child's head pulled off by injudicious traction. Pull the body downwards and backwards until the head has rotated around the promontory, when the forceps may be applied and delivery completed in the line of Carus's curve. The use of forceps in the fore-coming head is not based on sound mechanical principles in contracted pelves, except in minor degrees of deformity. When ordinary delivery is impossible, with no hope of saving the child, perforation of the head and escape of a few ounces of brain matter allows the skull to collapse, and delivery is comparatively easy.

Prof. Goodell said that he had been staggered by Boudin's experiments until he found the error in them. A pelvis was used with a movable promontory made to project by a screw. The pelvis itself was not a normal one, and every turn of the screw converted it more and more into a uniformly contracted pelvis, and not into the flat pelvis. Again the traction was straight; being made of pulleys attached to the wall, it allowed of no rotation. In kidney-shaped pelvis the promontory is rounded; in these experiments it was sharp. In reply to Prof. Lusk, he said the forceps were of advantage in many cases not mentioned, but he had confined himself to the conditions named in the paper.

### RELATION OF CONVULSIONS TO HIGH TEMPERATURE.

A paper was read "On the Management of Convulsions in Children, depending upon a High Temperature of the Body," by T. K. Holmes, M. D., of Chatham, Ontario, Canada.

The writer sketched the various causes of eclampsia in children, and believed that in many cases the convulsions are directly attributable to the effect of high temperature on the nervous system. The following propositions have been confirmed by experience: that extreme nervous susceptibility of children under ten years of age, predisposes them to convulsions; that bodily temperature of 103 (F.), and over, tends to produce them; that their severity bears a direct ratio to intensity of fever; that they are most apt to occur at the onset of the morbid attacks; and that whatever reduces temperature arrests or modifies the muscular spasms.

He referred to the indications for treatment in these cases, as suggested by the propositions just given, and stated that these can be accomplished more readily and safely by external than by internal medication—heat, which exhausts the power of the heart, or remedies which weaken cardiac power, being generally inadmissible. The bath being a thoroughly efficient substitute, should be resorted to in such cases. Several cases treated by the author were mentioned to show that in simple pyrexia, without zymotic influence, convulsions were caused by heat and promptly controlled by the bath. Tepid water is at first used to prevent alarm or shock, gradually reducing, by adding ice water, to about 60°, and until the axillary temperature becomes nearly normal, the patient being then taken out and put in blankets. The temperature of the body is not to be reduced in the bath to below the standard of health. This treatment is also available in diseases attended by high temperature, without convulsions. Other indications require treatment in convulsions, as the bowels, etc.

The Chairman said the rise in temperature was due in these cases to nervous irritation or blood poison. It would be interesting to know whether blood poison or hot blood, acting on nerve-centres, produced convulsions; but with rise of temperature there is unquestionably increasing liability to eclampsia. In some cases convulsions occur without material elevation of temperature, as in uræmic convulsions after scarlatina. He thought that scarlatinal patients could be dipped into the cold bath. He had for a long time used sponging with acetic acid in such cases. The credit of introducing cold bathing in fevers belongs to Dr. Parry of Liverpool; it had been dropped and temporarily forgotten, when it was revived by the Germans.

Dr. Rochester, of Buffalo, said it was fashionable to attribute increased tissue change, etc., to excessive heat, but thought other meteorological influences might be at work. When the sky is clear and temperature very high, cholera infantum is not so prevalent as when the air is loaded with moisture and the sky cloudy, though the thermometer may be lower.

ENUCLEATION OF OVARIAN CYSTS.

Dr. Julius F. Miner, Buffalo, N. Y., by invitation read a paper on the "Enucleation of Ovarian Cysts."

He stated that the enucleation of ovarian cysts was advocated by him seven years ago. It has been well received, but he thought his operation was not fully understood. After stating the relations of the ovarian cysts to the peritoneum and vessels, and the facility with which the cyst-wall is cut open, the tumor readily rolling out, he said that the fear of hemorrhage was unfounded, as the vessels moving from the peritoneal coat are capillary, and oozing is readily checked. Bleeding is more likely where the clamp is used and vessels cut across. A dry napkin to the collapsed cyst-wall checks the oozing, larger trunks are closed by torsion or ligature, and the contracted membrane is restored to the abdomen, giving no further trouble. If the operation fails, the clamp, cautery, or ligature may be subsequently applied. The diseased portion is removed, the living part returned; there is no pedicle outside, no need for drainage. Manipulation is no more difficult than separation of cystic growths from cellular tissue elsewhere.

(The paper contains the notes of several successful cases of enucleation of simple and multilocular cysts.)

The Chairman said no rule could apply to all cases. In some, enucleation was the only operation; he had been accustomed to regard it as a *dernier resort.* He does not hesitate to tie the pedicle with whipcord and return into the abdomen; he had seen no bad result from the ligature, but thought the clamp gives greatest security.

Dr. Jas. P. White, of Buffalo, said where the pedicle is large and flat we cannot enucleate satisfactorily; where there is bleeding the hot iron is useful to sear the stump; it leaves less in the abdomen for absorption, and pregnancy occurred in his practice as often after the operation as he had lost patients.

Dr. E. R. Peaslee, of New York, did not think the vessels of ovarian tumors are capillary, as in some polycysts serious bleeding ensued. He had lost one patient by hemorrhage after enucleation, a vessel deep in the pelvis being broken off that could not be secured. He saw no objection to applying ligatures. In small cysts there is no advantage in enucleation, but he would adopt it in cysts adherent to the liver. He generally cut the ligature close, and had only one case where the pedicle sloughed. With this exception he never in these cases lost a patient from the ligatures.

Dr. G. Kimball, of Lowell, had used enucleation once, but in other cases he regretted not doing so. He generally uses a clamp, but adapts the treatment to the case.

Prof. Simpson agreed with this last remark. Best results would probably flow from cases where the abdomen is closed, and nothing left in its interior to cause irritation or future trouble.

Prof. Theophilus Parvin had had two cases of enucleation; one complicated by pregnancy, had considerable bleeding checked by flannels dipped in hot water and freely applied. There is less risk in clamping than in returning large raw surfaces into the abdomen. Enucleation was valuable, but was not the only plan of treatment.

Dr. Miner said he found the vessels running in sulci, and avoids them in enucleating. Where there is bleeding he resorts to torsion, but enucleation did not interdict subsequent use of hot iron, ligature, or clamp.

*Wednesday, September 6th.*—Dr. Barnes presiding. Dr. W. L. Atlee, Reporter, read his paper on the

TREATMENT OF FIBROID TUMORS OF THE UTERUS.

The subject was treated mainly from the standpoint of personal experience. The paper inquired into two general subjects : I. Tumors usually accompanied with hemorrhage, embracicg (*a*), fibroids occupying the vaginal canal; (*b*), fibroids within the cavity of the uterus; (*c*), interstitial submucous fibroids; (*d*), interstitial fibroids proper; (*e*), recurrent fibroids. II. Tumors usually not accompanied with hemorrhage, including (*a*), interstitial subperitoneal fibroids; (*b*), sessile peritoneal fibroids; (*c*), pedunculated peritoneal fibroids; (*d*), interstitial cervical fibroids; (*e*), myomatous degeneration of the uterus; (*f*), fibrocysts of the uterus. He considered the best mode of treatment, both surgical and medicinal, as the removal of tumors *per vias naturales*, by abdominal section, the propriety of extirpating a fibroid uterus by either of these methods, and an inquiry into the several agents which are supposed to control the growth of fibroid tumors.

Dr. J. L. Atlee, of Lancaster, Pa., stated that, through an error of diagnosis, abdominal section had been performed in his practice in two cases of fibroids, followed by recovery.

In one the tumor was sessile, springing from the fundus; hemorrhage took place on one side, under the cyst-wall, giving a sense of fluctuation, and forcing the tumor into a half-moon shape. The growth, weighing 12 lbs., was removed, and a clamp applied. In the other case there was ovarian cyst on right side and a sub-peritoneal uterine fibroid (6 lbs.) in the left; both pedicles were included in the same clamp. He thinks this operation will soon be considered as legitimate as ovariotomy.

Dr. Alexander Dunlap, of Ohio, had noticed sub-peritoneal fibroids stopped growing after attaining a certain size—sometimes even receded. He described the ingenious plan for drainage outside the peritoneal cavity already alluded to in the *Record* several weeks since. He reported two cases of abdominal section for uterine fibroma; both died from want of drainage.

Dr. Kimball, of Lowell, Mass., was pleased with the suggestion just made, and with the general propriety of letting these growths alone. He reported a case of abdominal section for supposed ovarian cyst, which proved to be fibroma. The organ was extirpated, and a silk ligature applied. He was sent for six months later to remove the ligature, but found it firm; eventually it slipped inside, and gave no further trouble. The woman had gained 60 lbs. since the operation. He would not operate where the entire organ was involved and encroaches on the vagina, from fear of hemorrhage or exhausting suppuration. Instead of ligating the stump, he prefers the ecraseur, and sears the stump with the hot iron. Very few cases authorize extirpation of the uterus. He had not seen any result from muriate of ammonia or ergot in subperitoneal fibroids, but one case got entirely well after a year's treatment by iodide of potassium injections into the uterus, three times a week (grs. viij.–x. to ℥ i.).

Dr. Peaslee, in a case of uterine fibroma not attended by any symptoms, unqualifiedly recommends muriate of ammonia; where the tumor is interstitial, Squibbs's fluid extract of ergot (gtt. x.–xij., t. d.). In hemorrhage there is no better remedy than ergot and chloride of iron. In a variety of polypus growing from the posterior wall of the cervix, projecting into the canal, and perhaps into the vagina, there is always serious hemorrhage from the pedicle. In such cases he uses the ligature and antiseptic washes. In the negro race multiple fibroids are so numerous that in a home in New York he is informed they are invariably found in cases over forty years of age. In cases of doubt in diagnosis, rectal exploration is justifiable. He had restored a retroverted gravid womb, at the fourth month, by its aid. In the discussion fibrocysts should be separated from fibroids proper; the former generally spring from the posterior wall of the uterus and drag the organ upward, elongate the organ and bend it forward; this does not occur in simple fibroma, and is a diagnostic point.

Dr. H. Lenox Hodge, of Philadelphia, said: The best means of treating hemorrhage from uterine fibroma is to lift up the uterus and keep it in good position with an appropriate pessary. This treatment, introduced by his father, is followed by lessened bleeding, and in some cases by disappearace of the tumor, without internal medication.

Dr. C. B. King, of Pennsylvania, reported one case of operation by abdominal section and the clamp, with recovery.

Dr. Chas. Shepard, of Michigan, lost a case by not operating, and saved a second case by abdominal section and the ligature.

Dr. George Sutton, of Indiana, read notes of one subperitoneal and one submucous fibroid cured by ergot hypodermically, and had one fatal case.

Dr. E. H. Trenholme, of Montreal, reported two cases of successful laparotomy, one fibro-cystic and the other fibroid.

The Chairman said that all agree that this operation should not be resorted to unless life is in danger, but in an extremity we are bound to extend any hope of relief. He had lost three cases within a year by attempting to remove the tumor through the vagina, and is extremely shy of operating unless he can remove every particle of the growth. Septicæmia occurring, may be remedied by frequent antiseptic injections and administration of quinia by the mouth.

THE THREE IMPORTANT OBSTETRICAL INSTRUMENTS.

Three obstetrical instruments were exhibited by the Secretary, sent by Prof. Lazerwitch, of Kharkoff, Russia, with a folio containing photographs and descriptions of their application. They consisted of a pair of short forceps, fitting together in a peculiar way; a double blunt hook, with an eye in one extremity, by which a fillet could be passed; and a peculiar cephalotribe and tractor, which to the Chairman appeared, in principle, to be an old English instrument revived. A vote of thanks was returned to Prof. Lazarewitch.

*Thursday, September 7th*, 1876.—Prof. Simpson in the chair. Prof. Wm. T. Lusk, Reporter, read a paper on the

NATURE, CAUSES, AND PREVENTION OF PUERPERAL FEVER.

Looking on puerperal fever as a generic term, he examined into its different varieties, making a distinction between non-infectious forms. The non-infectious form was the result of traumatic injuries, old peritoneal adhesions, disregard of hygienic precautions, and mental influences; the infectious form was a septic disease. Local lesions were the usual, though not the necessary point through which the poison entered the system. He then inquired into the relations of bacteria to puerperal fever, the influence of erysipelas, scarlatina, diphtheria, etc., upon the puerperal state; and atmospheric influences. In studying the causes, he made deductions from civil and hospital statistics and private practice, and gave rules for prevention based on our knowledge of causes.

Dr. H. P. Yeomans, of Canada, remarked that lactic acid in the circulation would cause endocarditis, with deposits of fibrin on the valves; in such cases it is not necessary to suppose the presence of bacteria.

Prof. Goodell thought statistics of death from puerperal fever were unreliable in private practice, as physicians disliked to give certificates for it, and often gave instead one of the phenomena, as pleurisy or pneumonia, as the cause. This is one reason why the death rate in Maternities from this cause is relatively high. Of 1,021 patients treated at the Preston Retreat, there had been only three deaths from puerperal fever; not one case of pelvic abscess. He isolates the patients as much as possible. In incipient cases he gives enough morphia or opium to subdue the pain; quinia is a favorite remedy and alcohol given largely as an antiseptic and antipyretic, and to sustain the heart, as there is tendency to fatty degeneration.

Dr. Thos. W. Gordoni of Ohio, in a large practice, had seen only two cases of puerperal fever, but had seen many incipient cases arrested by ergot, quinia, and alcohol.

Prof. Byford said, cases of puerperal fever belonged to three principal classes; those arising from within the patient or her immediate surroundings; those occurring in the same neighborhood, endemic cases, the cause being zymotic, as in hospitals and poor neighborhoods. When this form prevails in hospital, with ordinary precautions he does not dread carrying the disease among his private patients. The third class of cases occurs under peculiar epidemic influences, as during prevalence of the black tongue in Indiana, in 1839, when in the first part of the epidemic all the puerperal cases died. There was no lack of ventilation or cleanliness; they were not communicated, as they occurred miles apart in the practice of different physicians, and in cases unattended by physicians. There must have been a wide-spread epidemic influence.

Dr. J. L. Atlee quoted three cases where puerperal fever was communicated from erysipelas.

Dr. Jas. P. White testified to the contagious nature of the disease, and its connection with erysipelas. No physician has a right to go from a case of erysipelas, or of puerperal fever, and attend a case of confinement.

Prof. Trenholme had five cases of puerperal fever, all of which recovered under careful treatment and large doses of morphia. Of six hundred cases of obstetrics, he had lost but one from any cause whatever.

Dr. Robt. Burns, of Philadelphia, recommended venesection in cases that would bear it, and internally, opium, gr. i., calomel, gr. ss. hourly, and a poultice to the abdomen.

The Chairman said, in all the works on the subject error creeps in when they discuss puerperal fever. The chapter on this subject should be entitled "Fevers of the Puerperal State," and extend from simple mastitis to those accompanied by poisoned blood. Many cases called puerperal fever were typhoid fever, occurring in the puerperal state, and more rapidly fatal on account of lessened power of resistance. Apart from fever caused by special poisons, which might suggest a division of cases into typhoid puerperal fever, scarlatinal puerperal fever, and those due to erysipelas, measles, etc., others closely resemble the fever following surgical operations. It would be interesting to inquire if soldiers wounded early in the fight, while fresh and vigorous, were less liable to surgical fever than others wounded in the same battle after hours of fatigue and exhaustion. An analogy exists here between such cases and the puerperal condition, so far as relates to dangers to which puerperal women are exposed from short and long labors.

We should study all the sources of this disease that we can find in our daily rounds. Most of the germ destroyers are acids—carbolic, sulphurous, salicylic, etc. There is nothing better to disinfect the hands than common vinegar; after extraordinary exposure, the clothing should be exposed to sulphurous acid fumes.

Prof. Lusk stated that masses of bacteria had been found on the cardiac valves in cases examined. He used Labarraque's solution to disinfect his hands, washing them to the elbows, perhaps twenty times a day during an epidemic. He had never carried the disease from hospital to private patients.

Dr. Fred. Semelender then read a paper on

ELECTROLYSIS, ESPECIALLY FOR THE CURE OF OVARIAN CYST.

After some general remarks on electricity, he gave an account of the effects of electric currents on solutions of salt or albumen, stating that the positive and negative pole act somewhat differently, and that from this circumstance different indications may be derived. He then passed to the cure of ovarian tumors by electrolysis, and gave a short statement of six cases he had successfully treated. The method is really new, though cases were published in 1859; they were long forgotten; the profession took no notice of them. No inflammation, no pain, no adhesions were caused; his patients were never put under chloroform, nor confined to bed. The cures required from one to five months; the patient is not deprived of one of her most interesting organs. He concludes emphatically: It is a case of conscience to try electricity, before ovariotomy is resorted to, as a patient will not find herself in a worse condition for the operation if electrolysis prove unsuccessful, while a little delay is an insignificant evil in comparison to the risks of ovariotomy.

*Friday, September 8th.*—Prof. Simpson in the chair. On motion, it was decided to have all the papers read *seriatim* before discussion.

Dr. Simon Fitch, of New York, read a paper on

"PARACENTESIS, ASPIRATION, AND TRANSFUSION."

It considered: 1. Paracentesis as it was, with the old trocar; 2. Paracentesis and aspiration as they are with modern instruments; 3. Paracentesis, aspiration and transfusion, as they should be with available apparatus. The following conclusions were given: 1. The old trocar, with big head and split canula, should be esteemed a relic of the past. 2. Modern trocars chiefly used are, either the pointed single tube, too often used in aspiration without a guard to cover the point, which is very dangerous; or the same guarded by a canula on the outside, which if thick is uncertain to enter; if thin, is only somewhat less hazardous than the point itself. 3. The trocar of the future should be easy of insertion, harmless when inserted, safe as a probe or sound, competent for the free passage of fluids, adapted to the aspirator, and certain to have a wound ready to heal. A series of instruments under the general name of dome-trocar was described by him, and its mode of application and advantages explained at some length.

It is said by Dr. Fitch to be adequate to make transfusion and other critical operations simpler, quicker, and safer, and so advance the efficiency and safety of paracentesis, aspiration, and transfusion of the future.

UTERINE HEMORRHAGE AND INVERSION.

Dr. E. H. Trenholme, of Montreal, then read a paper on "Uterine Hemorrhage," and Dr. James P. White, of Buffalo, one on "Chronic Inversion of the Uterus." Both were interesting and valuable contributions.

RETROVERSION OF THE GRAVID UTERUS.

Dr. T. F. Rochester, of Buffalo, reported a case of retroversion of the gravid uterus, between the fourth and fifth months. So completely was the organ capsized that it was impossible to find the os uteri. There was the usual hemorrhage from the bladder. It was found or judged impossible by position and manipulation to replace the womb. It was decided, on consultation with Prof. James P. White, to puncture or tap it, through the wall of the everted fundus, with a capillary trocar. This procedure was followed by the happiest results; in three days thereafter a fœtus nine inches long was expelled, and the mother recovered without an unfavorable symptom. Two months afterwards the womb was replaced with Simpson's sound, and retained *in situ* by a hard rubber cradle-shaped pessary. The patient continues perfectly well, but has not since become pregnant.

Dr. John L. Atlee cautioned against puncturing every retroverted gravid uterus. He recalled a case retroverted by full bladder easily restored after catheterization and which went on to full term. He had seen only one case of inversion in fifty years. He was summoned in consultation in an acute case, but found her in a condition of collapse that forbade operation; after advising opium in full doses, he left. Much to his surprise he heard some time afterwards that the woman did not die, but was again pregnant. He did refer to this as spontaneous reduction, but not purely natural; as the woman was again pregnant, the *modus operandi* was left to individual speculation.

Dr. W. J. Heddens, of Missouri, has reduced two recent cases of inversion with the air-bag.

Dr. W. L. Atlee reduced in ten minutes an inverted uterus of several months' standing, with the blunt end of the large dome-trocar of Dr. Fitch. He reported two other cases of retroversion of the gravid uterus caused by distended bladder, both easily restored; one went to full term; the other, complicated by sphacelus of the bladder, miscarried.

### A NEW CLAMP.

The Secretary exhibited a new clamp for ovariotomy, by Dr. Alexander Strubel, of Dresden, consisting of two sickle-shaped segments of steel drawn together by a screw, to secure the pedicle.

After passing a vote of thanks to Dr. Barnes and Prof. Simpson for their services in the chair, the section adjourned *sine die.*

### SECTION OF DERMATOLOGY AND SYPHILOLOGY.

*Monday, September* 4, 1876.—The Section was organized, Prof. Jas. C. White, of Harvard University, taking the chair, Dr. Arthur Van Harlington, of Philadelphia, Secretary.

### VARIATIONS IN SKIN DISEASES IN DIFFERENT COUNTRIES.

A paper on "Variations in Type and in Prevalence of Diseases of the Skin in Different Countries of equal Civilization" was read by James C. White, M. D., professor of dermatology in Harvard University, Reporter. This was based on statistics drawn from reports of leading dermatologists of Europe and America, embracing 50,000 cases from abroad, with 12,000 observed in this country.

As a result of examination and comparison of these statistics, he deduced certain conclusions embraced in a series of propositions, as follows:

1. Certain obscure affections, the etiology of which is little if at all understood, even in those parts of Europe to which they are mostly confined, may be regarded as practically non-existent among us. Of such are purigo, pellagra, and lichen exudativus ruber.

2. Certain diseases directly connected with and dependent on poverty and habits of personal uncleanliness are less prevalent in the United States than in those parts of Europe of which we have sufficient statistical information for comparison. Examples of this class are the animal parasitic affections especially.

3. Some cutaneous affections of grave character, dependent on, or a part of serious constitutional disorders, are of less frequent occurrence and of milder type amongst us than in Europe in general, or those parts of it where they are endemic. Lupus, the syphilodermata, and leprosy are the most marked instances of this class.

4. Certain disorders of the skin, especially those connected more immediately with its nervous system, are apparently more prevalent with us than in Europe. The most notable examples of the former are: seborrhœa, acne, and possibly heat rashes; of the latter, herpes, urticaria, and pruritus.

Considerable discussion ensued regarding some of these points. Dr. Duhring, of Philadelphia, thought that massing the figures from the three cities, Boston, Philadelphia and New York, gave a fallacious impression with respect to diseases in any one of these cities.

Dr. Bulkley, of New York, replied that the same discrepancy would be found to exist between the statistics of successive years in the same institution. He thought by collating the figures derived from reports of these localities, extending over a series of years, as the reporter had done, a very fair idea could be obtained of the prevalent skin diseases of the country.

Some discussion also took place on the use of the term morphœa by the reporter, and the relations borne by this disease to other affections sometimes called by the same name, as Addison's keloid, linear atrophy and scleroderma. Various opinions were expressed by Drs. Bulkley and Duhring, and by the reporter, the conclusion finally reached being that the term morphœa had been used in the sense generally understood by American dermatologists. The reporter's propositions were then taken up one by one.

On the subject of some of the affections mentioned, as prurigo and leprosy, no difference of opinion seemed to exist relative to the occurrence of these affections now in America. As regards the latter, Dr. Bulkley related a case which had come under his observation, occurring in a person of healthy American parentage, who had never been sixty miles away from New York. Cases were alluded to by the reporter as occurring in some of the Southern States, as well as the well-known leper colony in Nova Scotia. As regards the severity of syphilis as it is found in this country, the different members of the Section seemed to have had a different experience. Dr. Duhring said he was quite sure he had seen as large a proportion of very severe cases of this disease in Philadelphia as he had seen abroad.

The various propositions presented by the reporter were finally passed, it being placed on record, however, that the sense of the Section regarding the third proposition was understood to be, that lupus vulgaris is of a milder type and less prevalent in this country than in Europe; that leprosy is not sufficiently known to warrant an expression of opinion; and that, upon the subject of the syphilodermata, the Section differ from the reporter.

Dr. Bulkley offered the following additional proposition, which was adopted by the Section: The type of certain acute congestive and nervous diseases of the skin is more severe in this country than abroad.

The chairman called attention to a paper, entitled "Verugas, a Disease peculiar to Peru," by Dr. Ward, of Lima, which had been sent to the Section. On motion, the paper was referred to a committee.

*Tuesday, Sept. 5th.*—The Section met at 2 o'clock. Lucius Duncan Bulkley, M.D., of New York, Reporter, read a paper on the question,

ARE ECZEMA AND PSORIASIS LOCAL DISEASES, OR ARE THEY MANIFESTATIONS OF CONSTITUTIONAL DISORDERS.

He considered the nature of the eruption in constitutional disorders affecting the skin, as in the contagious fevers, syphilis, etc., and also the characteristics of local diseases; also the microscopic anatomy of eczema and psoriasis. He then studied the clinical history of eczema and psoriasis, according to age, sex, location, relapses, hereditary transmission, gouty and rheumatic symptoms, urinary disturbances, bronchitis, etc. He then examined into the clinical history of local diseases, epithelioma, verruca, parasitic, and mechanical diseases of the skin, etc., the effect of local and constitutional treatment, etc. The propositions brought forward by him aroused much opposition, and were debated one after another, the discussion lasting the whole afternoon. The debate seemed chiefly to turn upon the nature of eczema, and the various pathological theories connected with dyscrasia, diseases of the blood, and the cellular pathology, as bearing upon this affection.

The propositions, as finally adopted by the Section, were as follows:

1. Eczema and psoriasis are diseases *sui generis*, and are not to be confounded in any way with other states; as the former with artificial dermatitis, and the latter with the eruptions of syphilis, scaly eczema, or leprosy.

2. Eczema and psoriasis cannot own a double independent causation or nature, at one time local and another constitutional; but with other diseases, they may have a twofold cause, namely, a predisposing and an exciting.

3. Eczema and psoriasis, in many of their features, resemble the accepted constitutional diseases more than those recognized as local.

4. Local causes play an important part in the etiology of eczema; they are probably inoperative in psoriasis.

5. Certain relationships between psoriasis and epithelioma have been claimed, which require much further investigation. At present they are not established, and are no proof of the local nature of psoriasis.

6. No direct casual connection has yet been demonstrated between the scrofulous state and eczema and psoriasis.

7. Local treatment is often insufficient alone to remove the lesions of eczema and psoriasis, and cannot prevent or delay relapses.

8. The success of local treatment in eczema and psoriasis does not demonstrate the local nature of these affections.

9. Constitutional treatment alone and singly can cure many cases of eczema and psoriasis, and prevent or delay relapses in a certain proportion of cases; under constitutional treatment it is intended to signify every agency not properly placed among local measures.

10. The total weight of argument is that eczema and psoriasis are both manifestations of constitutional disorders, and not local diseases of the skin.

Prof. Rudnew, of St. Petersburg, then read a paper entitled

"WHAT IS THE DISEASE KNOWN AS LUPUS?"

by Dr. Woskrensky, of the Institution of Pathological Anatomy, St. Petersburg, under Prof. Rudnew's directions. The name was originally loosely applied, just as "sarcoma," "cancer," etc., were. The changes of the tissues in lupus have no signs by which it can be considered a separate and independent disease. It is merely a name applied to several species of tumors. From personal observations in facial cases, he considered that all the different varieties might be classed under three divisions: 1, a form which is simply a horny cancer, and incurable; 2, adenoma simplex, or confounded with the cancer; 3, granuloma syphiliticum, easily cured.

*Wednesday, September 6th.*—Freeman J. Bumstead, M.D., late Professor of Venereal Diseases at the College of Physicians and Surgeons, New York, Reporter, read a paper on

THE VIRUS OF VENEREAL SORES; ITS UNITY OR DUALITY.

The following propositions were stated by the reporter:

1. The virus of venereal sores is dual.

2. Venereal sores may be due to the inoculation of the syphilitic virus, and also to the inoculation of the products of simple inflammation.

3. These two poisons may be inoculated simultaneously.

The second proposition aroused a lively debate, the reporter being understood to express the view that there was no specific chancroidal virus. At the suggestion of the Chairman, the proposition was divided into two sections. The first of these was passed without discussion; but as to the second, all that portion of the original proposition which follows the word "virus," was, after long debate, rejected by the Section.

In connection with the second proposition, Dr. Bulkley offered the following, which was adopted by the Section.

The present state of science has demonstrated that suppurative inflammatory lesions resembling chancroids may be produced on different portions of the body by inoculation with simple pus from various lesions.

A paper by Dr. Drysdale, of London, bearing the same title as that of Dr. Bumstead, was then presented by the Secretary, and, on motion, was passed by title.

The Secretary then read a paper entitled

LEPROSY IN THE SANDWICH ISLANDS,

By Dr. F. H. Enders, Government physician. They believed it was introduced from China, but probably this was not so. The writer was unable to say positively whether it was a disease *sui generis*, or a form of syphilis, although he inclined to the latter view. He had seen over four hundred cases, all of whom except two had had syphilis. Two forms of leprosy were met with, the anæsthetic and the tubercular, cases of which were cited. Many interesting facts in its history, treatment, etc., were given.

SEBORRHŒA.

Dr. Heitzmann, of New York, read a paper on "The Treatment of Seborrhœa." A discussion upon the subject of this paper ensued, in which various members of the Section gave their experience in the treatment of the affection.

THE CONSTITUTIONAL TREATMENT OF SYPHILIS.

*Thursday, September 7th.*—The Section met at 2 o'clock P.M. A paper was read on "The Treatment of Syphilis, with Special Reference to the Constitutional Remedies appropriate to its various Stages; the Duration of their Use, and the Question of their Continuous or Intermittent Employment;" by E. L. Keyes, M.D., Adjunct Professor of Surgery and Professor of Dermatology in Bellevue Hospital Medical College, New York, Reporter.

The propositions presented were as follows:

Negative conclusions: views for which there would seem to be no foundation in fact.

1. Syphilis commencing mildly needs but little treatment, and does not require mercury.

2. Mercury given internally is necessarily debilitating.

3. Mercury is only useful in secondary syphilis.

4. Iodide of potassium is of considerable value in secondary syphilis.

5. Iodide of potassium is of no value unless preceded by the use of mercury.

6. Iodide of potassium acts by liberating mercury which has been lying latent.

The following positive conclusions, which in the present state of our knowledge may be affirmed, were then enunciated by the reporter, and laid before the Section.

1. Mercury is an antidote to the syphilitic poison, and of service in controlling all its symptoms in all, even the latest stages, of the disease; its power over gummata being least, and not to be relied upon.

2. Mercury in minute doses is a tonic.

3. Iodine cures certain symptoms of syphilis, but does not prevent relapses.

4. Mercury long continued uninterruptedly, so far as practicable in small doses from the time of earliest eruption, constitutes the best treatment of syphilis.

These propositions gave rise to much debate, the discussion for the most part including the question of continuous or interrupted methods of treatment. It was finally conceded that the statement was of a scientific nature, and suggested the object to be aimed at, rather than that which is generally attainable.

---

### SECTION OF BIOLOGY.

*Monday, September* 4.—The Session met, Prof. S. E. Chaille, of New Orleans, in the chair; Dr. James Tyson, of Philadelphia, Secretary.

### MICROSCOPY OF THE BLOOD.

Prof. Christopher Johnston, Reporter, read a paper on this subject, in which he inquired into the original source of blood in vertebrates; and the elements of blood in them. The normal elements having form were exclusively considered, from two points of view: that of anatomy and physiology, and of medical jurisprudence. He discussed the genesis of corpuscles; the form of colored corpuscles, and their structure; leucocytes; the size of colored corpuscles, and their enumeration, and the relation of the colored blood-corpuscles to medical jurisprudence.

The discussion which followed was almost a repetition of that had at the recent American Medical Association meeting, and was confined to the medico-legal application of our knowledge of the size of the red blood-discs. The reporter took the ground that it was impossible to identify by their size the red discs of human blood from those of other mammals nearly related in size.

The chief point at issue was stated by Dr. J. G. Richardson, of Philadelphia, to be as follows:

He said, if his memory served him correctly, Prof. Johnston had mentioned that when Dr. Woodward showed him a series of specimens of blood-corpuscles, including samples of guinea-pig, dog, and man's blood, he found it impossible to discriminate one from the other. This result was exactly what he would have anticipated, but he would like to ask Dr. Johnston whether, in case Ananias (by way of complete antithesis to Dr. W.) offered him a slide for inspection, and concealing the label, told him it was sheep's blood, he could not, if the corpuscles proved on measurement to be $\frac{1}{3200}$ of an inch in diameter, say most emphatically, "Ananias, you lie?"

Dr. Didama, of Syracuse, New York, was inclined to agree with Dr. Richardson's general views on the subject.

Dr. Tyson, of Philadelphia, thought we might go so far as to say that such blood was more likely to be the blood of man than of an ox, or sheep, or pig, but would not be willing to say positively such blood was derived from a human being rather than from one of the animals mentioned; he could not but think, with his present knowledge, that there might be circumstances in the drying, age, or texture of the fabric on which they lay, which could make it impossible to distinguish human blood-corpuscles from others, even as much smaller as those of the ox and sheep.

Dr. Joynes, of Richmond, Va., inquired of Dr. Richardson how far the original character of corpuscles, dried on cloth or similar textures, could be restored by maceration, and expressed the fear that difficulties of this kind might cause error.

Dr. Richardson replied that it had been pretty well determined, that while corpuscles may shrink from five to ten per cent. in their original diameter in drying, it had not been found that they swell or increase their diameter by soaking. In conclusion, he exhibited under a Holmes Class microscope a red blood disc from the Amphiuma tridactylum, the hæmatocrystallin of which had crystalized in the upper end of the corpuscle, whilst the nucles occupied the lower extremity, leaving the membranous corpuscle (cell wall) hanging in folds like a half flaccid balloon between them.

EXCRETORY FUNCTION OF THE LIVER.

*Tuesday, September* 5.—Prof. Austin Flint, Jr. of New York, Reporter, addressed the Section on this subject. It was an interesting re-statement of the well-known views of the reporter, that cholesterine is an excremantitious substance, bearing the same relation to the liver that urea bears to the kidneys; is discharged in the bile into the small intestine; is transformed during digestion into another substance (stercorine), and, as stercorine, exists in the fæces.

Prof. Dalton, of New York, then followed in some interesting remarks on the "Importance of Physiological Chemistry in Physiology," and alluded to Dr. Flint's investigations as being in this direction. He gave also a history of the succession of discoveries connected with this subject, beginning with the finding of cholesterine in gall-stones, and culminating in Dr. Flint's elaborate investigations and discoveries.

Dr. Flint's views were generally concurred in.

*Wednesday, September* 6.—Prof. Rudnew, of St. Petersburg, Russia, read several papers appropriate to this Section, from investigations made under his direction:

One by Dr. Jantchich, on the "Termination of the Nerves in the Human Skin."

One, by the same, on the microscopical anatomy of the nervous apparatus of the bronchia and lungs; and two other papers on kindred subjects; but the descriptions being minute, although interesting, do not admit of the possibility of an abstract.

PREVENTION OF FUNGUS GROWTHS.

Dr. Joseph G. Richardson, of Philadelphia, then read a paper on "Fungous Growths in Solutions for Hypodermic Medication, and their Prevention by Salicylic Acid."

He stated that the toxic property of salicylic acid, superior to most powerful antiseptics, suggested its employment to prevent growth of tufts of fungi in morphia solutions, and in order to test its precise value, he devised a series of experiments, in which different quantities of salicylic acid were added to the usual Magendie's solution, other portions of the same fluid being allowed to remain unprotected for the sake of comparison. His results showed that even in summer morphia solution remained for over a month almost free from fungous growths, when it contained one grain of salicylic acid to the ounce of liquid; when the quantity of acid was doubled, the fluid was preserved entirely free of fungi. He also proposed the use of salicylic acid as a preservative for medicinal solutions of other alkaloids or salines, and for vegetable infusions.

In reply to a question of Prof. Dalton, Dr. Richardson said that although he had repeatedly injected his own arm with solutions containing fungi, he had never experienced any inconvenience either by formations of abscess or by general symptoms attributable to it.

*Thursday, September 7th.*—Prof. Harrison Allen, of Philadelphia, Reporter, read a paper on the "Mechanism of Joints," which contained many novel suggestions and facts. It was shown, for instance, that when the ball is supported by the socket, as at the temporo-maxillary articulation, motion is suggested. In illustration, the etiology of fracture and dislocation was examined. Articular surfaces were studied in three classes: axial, actinic, and lateral, and these terms defined with examples. A correct knowledge of the symptomatology and treatment of diseases of the joints was shown to be dependent on a true conception of the complex nature of articular surfaces.

Prof. Dalton said the paper contained much that was new to him, and explained some points which had long been unexplained, and that the same reasoning might also be extended to the ligaments. While dissecting joints as a student of medicine, he had been struck with the fact that in flexing and extending the limbs, it was impossible to say when certain parts were put upon the stretch and when they were not. He thought this might be explained by supposing the internal surface of the capsule to be made up of innumerable facets brought successively into action, just as Dr. Allen had shown to be the case with the curved articular surfaces of the bones.

With the exception of several papers on Friday, presented by Prof. Rudnew, of St. Petersburg, from Russian investigators, on "Sclerosis of the Arteries," "Cancer in the Lymphatic Glands," etc., no other paper of importance was discussed in this section.

---

### SECTION OF SANITARY SCIENCE.

*Monday, September 4th.*—The Section had assigned to it as Chairman, Dr. Stephen Smith, of New York, and Dr. Ezra M. Hunt, of New Jersey, as Secretary; Dr. Billings, U. S. A., and Dr. Baker, of Michigan, as Vice-Presidents. In the absence of Dr. Smith, Dr. Billings presided.

DISEASE GERMS.

The session was almost entirely occupied by the paper of Dr. T. E. Statterthwaite, of New York city, Reporter, on "Disease Germs." It presented, in a concise way, the vegetable germ theory, the bioplasm theory, and the physico-chemical theory. A fair exhibit was given of the arguments adduced in support of each of these. The author recounted his own experiments and those of Dr. Edward Curtis, of New York city, and presented diagrams of the various appearances under the microscope, and specimens of vacuum-tube experiments. Right emphasis was laid upon the point that motion does not always indicate life. Fluids were shown still to be poisonous after such filtration as would separate vegetable organisms.

The following general topics were discussed: The agency of minute vegetable organisms in fermentation and putrefaction; epidemic diseases of plants and animals in their relation to such organisms; rapid multiplication of bacteria *pari passu* with the rapid spread of disease manifestations throughout the system; constant ratio between the most active changes in so-called septic diseases, as pyæmia, erysipelas, and puerperal fever, with numerical increase in bacteria at points involved; can any strictly chemical substance be a fever producer; bacteria and disease poisons, their capacity for successfully maintaining active properties; inoculation of bacteria in healthy tissues.

The following special topics were also considered :

1. Bacteria: their classification, diagnosis, and appearances under varying conditions.

2. Poisonous fluids of infective diseases, their physical properties and the solid particles contained in them.

3. The value of vacuum-tube experiments.

4. How far are the bioplasm, or physico-chemical theories competent to explain the spread of infective diseases?

5. Poisons of special diseases, as cholera, small-pox, carbuncular diseases of men and animals, typhoid and relapsing fevers, and diphtheria, are not proved to be dependent on these minute organisms. The poison of diphtheria membrane, for instance, killed twenty out of twenty-seven rabbits, when no bacteria were present.

The paper was discussed by Drs. Henry Hartshorne, of Philadelphia, Hunt, of New Jersey, Billings, U. S. A., and others.

Dr. Billings preferred the term "minute vegetable organisms" to the terms bacteria, microzymes, etc., so often used, as these latter seemed to state a theory.

Dr. Hunt quoted Pettenkofer as saying, "what germs are, we do not know." The bacteria found in various infective diseases cannot be distinguished from one another, or from those of healthy persons. Billroth makes them all forms of one mother plant. The views of Klein and the hope of Simon that the contagion of enteric fever had been found, were alluded to, and also those of Oertel in reference to diphtheria. Dr. Hunt claimed that fermentation and putrefaction must be studied as separate decompositions, and that vegetable organisms were most frequently the effort of nature to dispose of the debris of disease, and so conservative—except where, from great accumulations, they might become themselves a source of evil.

Dr. Hartshorne expressed the view that we did not sufficiently recognize this compensating effort in nature; but still as there are venomous insects that have no conservative work, so there are such factors of disease.

The germ theory also came up prominently in the Surgical Section in connection with the antiseptic treatment of Prof. Lister. The Sanitary Section adopted the conclusions proposed by Dr. Satterthwaite after slightly modifying them, and the paper was recommended for publication.

The conclusions were as follows:

1. That so far as inquiry has been made into the nature of the active principles in infective diseases, it is probable that in a certain number the matter is *particulate* or *molecular* in form, and, in the instances named, in no sense a soluble substance.

2. That in regard to the *causes* of septicæmia, pyæmia, puerperal fever, erysipelas, and hospital gangrene, and in cholera, small-pox, the carbuncular diseases of men and animals, typhoid, relapsing fevers, and diphtheria, there is not satisfactory proof that they are necessarily connected with minute vegetable organisms.

3. That the real nature of these causes is still uncertain.

VITAL STATISTICS OF BUENOS AYRES.

A paper on this subject was offered by Dr. Rawson, of that city, and referred to a committee for examination. Abstracts were read from it the next day. It contained some interesting matter, but did not, amid the imperfection of registry, throw much light on the subject. It noted the fact that offal, sometimes to the amount of three hundred and ninety tons daily, was carried out of the city and burned. It insisted on the importance of looking to the health of native-born children in every country, as these are generally better members of society than immigrants. The frequency of trismus nascentium was noted, causing at times 6.5 per cent. of all the mortality. Parts of the paper were worthy of a place in the transactions, and it was referred for publication.

HOSPITAL CONSTRUCTION AND VENTILATION.

*Tuesday, September* 5.—The Section was occupied with the paper of Dr. Stephen Smith, of New York, Reporter, on these subjects, and with a detail of the more recent plans for the "Johns Hopkins Hospital," by Dr. Billings, of Washington.

Dr. Smith's paper was designed to show the very advanced position of that art at the close of the century 1776, the comparatively little progress made during the century 1876, and the present status of hospital reform. The position of the art in 1776 was illustrated by: First. The New York Hospital, a pavilion hospital, then recently destroyed by fire, which had been built according to plans brought from England by Dr. John Jones, and has not been surpassed in its essential features, especially in regard to the separation of the sick by large surface and cubical areas. Second. The report of Committee of French Academy of Sciences, 1788, on rebuilding the Hotel Dieu, giving in much detail the pavilion plan. These embodied in its highest state of perfection what is now recognized as the pavilion hospital. Little progress was made during the nineteenth century, except the building of the Lariboisiere, of Paris, on the pavilion plan, until the Crimean war, when the pavilion system was again revived by Miss Nightingale. While the reform of military and quarantine hospitals is all that can be desired, too little progress has yet been made in location and construction of civil hospitals. Propositions were submitted embodying latest suggestions of science and experience.

Dr. Billings, after a lucid explanation of the plans of the Johns Hopkins Hospital as at present suggested, and the reasons therefor, compared at length the different plans of ventilation, and showed why the fan was often more available and more adapted for combination with natural modes than the aspirating method. He drew attention to the tendency of air to cling to surfaces, and presented the results of some anemometer and other experiments as to heating and ventilation conducted within the past year at a hospital in Washington.

Many questions were asked, and considerable discussion ensued, in which Drs. Henry Hartshorne, Bowditch, Hewitt of Red Wing, and others, participated. Drs. Bowditch and Hewitt especially insisted that we depended too much on physicists and experiments, and that, after all, the sensations of the patients and the experience of the physician were more reliable. Others regarded mere sensation as unreliable, and only to be compared with other indications. The paper was referred to the Congress for publication, without formal adoption of its conclusions.

GENERAL SUBJECT OF QUARANTINE.

On Wednesday, September 6th, the paper on Quarantine, with particular reference to cholera and yellow fever, by Dr. Woodworth, Supervising Surgeon-General, Marine Hospital Service, Reporter, with its discussion, occupied the whole session.

After reviewing briefly the practice of quarantine in the past, and as at present administered, the mode of propagation of cholera and yellow fever were discussed with the view of arriving, as near as possible, at what precautions are necessary and what restrictions superfluous in the administration of quarantine.

The chief points were the practice and methods which should be pursued to secure greatest protection to public health against cholera and yellow fever with the least restriction upon commerce. In this connection were considered the want of prompt information to threatened ports of the shipment of passengers or goods from infected districts; the question of time as an element in quarantine; the value and practice of disinfection; the importance of municipal sanitary co-operation; and what may be gained by imparting to masters of vessels correct views of sanitary measures to be enforced by them in outbreaks of cholera or yellow fever on shipboard, etc.

The papers involved the chief laws of sanitation, of contagion, and of specifics of disease, and had material for the fullest discussion, in which Drs. Vanderpoel, Hartshorne, Murray, and others took part. Dr. Vanderpoel's personal experience was listened to with interest, and other speakers showed an intelligent acquaintance with the defects of ship regulations. Some discussion took place as to the relation of filth to disease, and the distinction was urged between filth as a nest, and filth as a creator of the disease germ, and so an originator of a specific disease.

With some slight modifications, the conclusions presented were adopted, and the paper highly commended for publication.

DISPOSAL OF SEWAGE.

*Thursday, September 7th.*—The Section was occupied with the subject of "The Disposal of Sewage." Dr. Henry Hartshorne, Reporter, read a paper setting forth the various plans for this object, giving preference to the water method and to soil filtration through drained land, so that value to crops and perfect conveyance to water-courses could be secured.

Dr. S. W. Gross presented a plan of sewer ventilation, suggested by a Philadelphia engineer.

Dr. J. G. Kerr, of China, spoke of Chinese methods. The chief idea in China was to dispose of offal above ground, while we sought to place it below ground. He believed epidemics were not, as a rule, so severe in Chinese cities, because they avoided soil saturation and soil pollution.

Dr. Johnson, of Salem, Mass., noted the evils arising from inflowing tide meeting outflowing sewage, and so often causing sewer pollution. The conclusions of the paper were modified so as not to give exclusive prominence to any one method of disposal.

UNIVERSAL PHARMACOPŒIA.

Dr. Squibb, of Brooklyn, read a paper on this subject, which, in the absence of a materia medica or pharmacy Section, had been assigned to preventive medicine. Mistakes arise from confusion in nomenclature. Dr. Squibb showed in what respects a universal pharmacopœia would be of service, and yet recognized such embarrassments as to preclude a hasty attempt in that direction. In the meantime he claimed that greater uniformity should be sought and unnecessary diversity of names be avoided.

PHARMACY AND MEDICINE.

Dr. Hunt, of New Jersey, also read a paper on the evils arising from separation of pharmacy and medicine. He claimed that pharmacy, like any specialty of medicine, should be a department rather than accessory, and should be replaced within the profession, and so held accountable to its ethics. It was ably discussed by Drs. Steiner, of Maryland, Squibb, Hartshorne, and others. All the papers were referred for publication.

WEIGHTS AND MEASURES.

*Friday, September 8th.*—Dr. Squibb presented an able paper on "The Metrical System of Weights and Measures." It showed the feasibility of the system, and the methods by which its adoption could be facilitated. Its adoption by two prominent nationalities already, indicates its more extended use, and this should be diligently encouraged.

Dr. Bowditch noted the fact that it had met with some opposition from a prominent scientist of Boston, and Herschel had also made some forcible objections to it. Any system which is more or less arbitrary can have real objections filed against it. But this works so well in practice, that few practical operators now dissent.

MEDICAL MISSIONS.

Dr. Kerr, of China, read an interesting paper on "Medical Missions," reference being had to sanitary and other regulations in china and other countries. The paper was discussed by Dr. Harris, of New York, and such parts of it as referred to medicine were ordered to be referred for publication. The section then adjourned *sine die.—Medical Record.*

